# Patient-Derived Xenografts Are a Reliable Preclinical Model for the Personalized Treatment of Epithelial Ovarian Cancer

**DOI:** 10.3389/fonc.2021.744256

**Published:** 2021-10-04

**Authors:** Jiayu Chen, Ying Jin, Siyi Li, Cui Qiao, Xinxin Peng, Yan Li, Yu Gu, Wei Wang, Yan You, Jie Yin, Ying Shan, Yong-Xue Wang, Meng Qin, Hongyue Li, Yan Cai, Yu Dong, Siying Peng, Lingya Pan

**Affiliations:** ^1^Department of Obstetrics and Gynecology, Peking Union Medical College Hospital, Chinese Academy of Medical Sciences & Peking Union Medical College, Beijing, China; ^2^National Clinical Research Center for Obstetric & Gynecologic Diseases, Beijing, China; ^3^Beijing IDMO Co., Ltd., Beijing, China; ^4^The Bioinformatics Department, Precision Scientific (Beijing) Co., Ltd., Beijing, China; ^5^Department of Pathology, Peking Union Medical College Hospital, Beijing, China; ^6^Department of Obstetrics and Gynecology, Beijing Cancer Hospital, Beijing, China; ^7^The Medical Department, Precision Scientific (Beijing) Co., Ltd., Beijing, China

**Keywords:** ovarian cancer, animal model, chemotherapy response, molecular biology, precision medicine

## Abstract

To generate robust patient-derived xenograft (PDX) models for epithelial ovarian cancer (EOC), analyze the resemblance of PDX models to the original tumors, and explore factors affecting engraftment rates, fresh cancer tissues from a consecutive cohort of 158 patients with EOC were collected to construct subcutaneous PDX models. Paired samples of original tumors and PDX tumors were compared at the genome, transcriptome, protein levels, and the platinum-based chemotherapy response was evaluated to ensure the reliability of the PDXs. Univariate and multivariate analyses were used to determine the factors affecting the engraftment rates. The engraftment success rate was 58.23% (92/158) over 3–6 months. The Ki-67 index and receiving neoadjuvant chemotherapy can affect the engraftment rate in primary patients. The PDX models generated in this study were found to retain the histomorphology, protein expression, and genetic alteration patterns of the original tumors, despite the transcriptomic differences observed. Clinically, the PDX models demonstrated a high degree of similarity with patients in terms of the chemotherapy response and could predict prognosis. Thus, the PDX model can be considered a promising and reliable preclinical tool for personalized and precise treatment.

## Introduction

Epithelial ovarian cancer (EOC) ranks first in mortality among all gynecologic malignancies. Owing to high rates of recurrence and chemotherapy resistance, the 5-year survival for advanced cases remains at only 29% (1–3).

Animal models can help to screen new antitumor drugs and optimize medication regimens (4, 5). Stable cell lines and cell line-derived xenografts (CDXs) have been used in drug screening tests, but they do not well reflect the patient state due to the lack of cancer heterogeneity and a tumor microenvironment; thus, approximately 88% of new drugs identified by CDXs fail in clinical research (6–8). Patient-derived tumor xenografts (PDXs), constructed by directly transplanting tumor tissue into immunodeficient mice, most resemble the original tumor, which can help clinicians better understand tumor behavior and facilitate therapeutic exploration (6, 9, 10).

PDXs were developed prior to CDXs. Rygaard et al. (11) first established a PDX model of rectal cancer in 1969, while the first CDX model dated back to 1972 (12). CDXs instead of PDXs were subsequently used as drug-screening tools by the National Cancer Institute (NCI) for a long time because PDXs require fresh samples, are more time consuming, and have a low engraftment rate. However, researchers have gradually realized the limitations of CDXs since the early 21st century and have rekindled their enthusiasm for PDX research. Moreover, the NCI announced that PDXs replaced NCI-60 cell lines as the most commonly used preclinical model in 2016 (8). PDXs have been successfully developed to model lung cancer, gastrointestinal cancer, breast cancer, and gynecologic cancer (10, 13–17).

Although some studies have already described PDX models for ovarian cancer (13), these studies used different protocols and included a limited number of patients from discontinuous populations, leading to obvious bias, and therefore do not well reflect true nature of EOC-PDXs. For example, the engraftment rate has varied among experiments, and few have investigated the reason for this difference (18–23). In addition, the chemotherapy regimen administered in PDX models is different from that administered in the clinic, so it is impossible to accurately compare the chemotherapy response between PDXs and corresponding patients (18, 19, 22, 24, 25). Moreover, an increasing number of studies have questioned the PDX model’s fidelity recently since it may show variation in the genome and transcriptome from the original tumor (26–28).

More evidence is needed before PDXs are applied broadly as a preclinical model for EOC. This study aims to develop PDX models from a consecutive cohort of EOC patients to identify whether PDXs could be authentic drug screening tools by comparing morphologic, molecular, and clinical manifestations between PDXs and the original tumors. Factors affecting the engraftment rates in EOC-PDXs were also explored.

## Materials and Methods

### Tumor Specimens and Experimental Animals

Between January 1, 2018, and October 30, 2019, 158 patients with EOC who had sufficient fresh cancer tissue samples from the primary lesion or peritoneal metastasis for research were enrolled consecutively and unselectively. Two oncologic pathologists independently confirmed all pathologies. The experiments were approved by the Institutional Review Board of Peking Union Medical College Hospital (PUMCH). Sufficient tumor specimens were immersed in serum-free RPMI 1640 media (Gibco, Cat# 11875-093) at 4°C and transplanted within 12 h. NOD-Prkdcem1Idmo-Il2rgem2Idmo (NPI) mice were purchased from Beijing IDMO Co., Ltd. (five mice per cage, specific pathogen-free) handled according to PUMCH’s Institutional Animal Care-approved protocol.

### Construction and Management of PDX Models

Female mice aged 6–8 weeks were anesthetized with 15 mg/kg Zoletil^®^ (Virbac) and 2.5 mg/kg Rompun^®^ (Bayer) by intraperitoneal injection. Tumor fragments 3 mm × 3 mm × 3 mm in volume were subcutaneously embedded into the flanks of NPI mice at one or two sites. Tumor volume was measured by a digital caliper (Volume = Length × Width^2^/2) twice per week, and the PDX model was considered established successfully (the “zero” passage, P-0) when the tumor volume reached 800 mm^3^. Then, the mice were euthanized, and the tumor tissue was harvested. Transplantation of P-0 tumor tissues to other NPI mice following the same method was employed to establish the first, second, third, etc., passages (P-1, P-2, P-3…).

### Flow Cytometry Analysis

Samples were cut into small pieces and digested with 0.25% trypsin. Then, the single-cell suspension was incubated with APC anti-human CD19 antibody and PE anti-human CD45 antibody. Leukocytes were identified as CD19+ (24) and CD45+ (25), and samples containing less than 1% leukocytes were qualified for further mouse-to-mouse transplantation.

### Histopathological Analysis

Hematoxylin–eosin (HE) staining and immunohistochemistry (IHC) were adopted to compare the morphology and protein expression of paired patient (PA) and PDX samples. Eight primary antibodies were used: PAX-8 (Abnova, Cat# PAB14858), CK7 (Abcam, Cat# ab154334), CK20 (Abcam, Cat# ab217192), P16 (Abcam, Cat# ab189034), P53 (Abcam, Cat# ab131442), WT-1 (Abcam, Cat# ab180840), ER (Abcam, Cat# ab3575), and PR (Abcam, Cat# ab191138). Sections were then treated with the secondary antibody (anti-rabbit IgG, Abcam, Cat# ab6120), followed by DAB chromogenic and hematoxylin staining.

### Molecular Analyses

#### DNA and RNA Library Construction

Genomic DNA from the PA and PDX tumors and from the peripheral blood mononuclear cells (PBMCs) collected from 10 HGSOC patients were extracted using the QIAamp DNA Mini Kit (Qiagen, Cat#51306). DNA (0.3–0.5 µg) was sheared into 200- to 300-bp fragments using a Covaris^®^ M220 ultrasonicator followed by repair and 3’ poly-A tailing. Then, adaptors were ligated to both ends of the fragments and amplified *via* polymerase chain reaction (PCR). DNA libraries were generated with the xGen Hybridization and Wash Kit (IDT, Cat# 1080584), followed by PCR amplification. Total RNA of PA and PDX tumors from nine patients was extracted using TRIzol™ Reagent (Invitrogen, Cat# 15596026). RNA (0.1–1.0 µg) was used to generate libraries using the Poly(A) mRNA Magnetic Isolation Module Kit (NEB, Cat# E7490L) and NEBNext Ultra II RNALibrary Prep Kit (NEB, Cat# E7770L). The DNA and RNA libraries were sequenced on an Illumina NovaSeq 6000.

#### Whole-Exome Sequencing Data Analysis

Only pairs with <3% N bases and >50% high-quality bases were kept for whole-exome sequencing (WES) analyses, which were then aligned to the human reference genome (Homo_sapiens_assembly19) using Burrows-Wheeler Aligner (0.7.17). The Genome Analysis Toolkit (version 3.8.1) was used to process BAM files to mark duplicates and local realignment around high confidence insertions and deletions, followed by BAM matching, which tests whether the matched PA tumors, PDX tumors, and PBMCs were from the same patient. Then, variant calling was performed through the pipeline developed by the TCGA MC3 project, which employed six callers to call substitution mutations and three callers to identify small indels. Only substitution mutations and indels supported by at least two callers were retained for further analyses. All mutations were retained for subsequent analyses if the position was ≥10× in both normal and tumor samples.

#### RNA-Seq Data Analysis

TopHat2 was used to align RNA-seq reads to the reference. DESeq2 was used to identify differentially expressed genes (DEGs) between paired patient specimens and PDX tumors after batch removal through Combat-Seq.

### Chemosensitivity Tests

#### Platinum Treatment of PDX Models

The P-1 to P-3 PDX models, bearing a tumor size of 100–200 mm^3^, were randomly assigned to the experimental group (*N* = 4) and control group (*N* = 4). For the experimental group, the PDX model administered the same chemotherapy regimen as the corresponding patient: (1) 30 mg/kg paclitaxel *via* intravenous injection every 4 days × 8 cycles; (2) 25 mg/kg carboplatin *via* intraperitoneal injection every 5 days × 6 cycles; (3) 3 mg/kg cisplatin *via* intraperitoneal injection every 3 days × 9 cycles; or (4) 5 mg/kg doxorubicin *via* intravenous injection every 2 days × 9 cycles. Tumor volume and body weight were measured every 3 days for at least 2 months after the first administration.

#### Response Calls

The platinum-based chemotherapy response of the PDXs was assessed with the modified Evaluation Criteria in Solid Tumors (mRECIST) (29). The best response was the minimum volume change percentage (10 days after the first administration), and the best average response was the average of that. Four response grades were described as follows: modified complete response (mCR), best response <−95% or best average response <−40%; modified partial response (mPR), − 95% ≤ best response <−50% or −40% ≤ best average response <−20%; modified stable disease (mSD), − 50% ≤ best response <35% or − 20% ≤ best average response <30%; and modified progressive disease (mPD), not otherwise categorized.

### Clinical Data Collection and Statistics

The patient’s clinical, pathological, and prognostic information was collected from the Hospital Information System of PUMCH. The pathological type and grade were based on the WHO classification and grading system (30), and the stage was determined according to the International Federation of Gynecology and Obstetrics (FIGO) staging system (31). Pathological types were reclassified into type I EOC and type II EOC. The definition of a platinum-based chemotherapy response followed the clinical guidelines: platinum sensitivity was defined as a progression-free interval (PFI) of more than 6 months; any other response was defined as platinum resistance. Progression-free survival (PFS) was defined as the time interval between the initial treatment and cancer progression.

Continuous and categorical variables were analyzed by Student’s *t*-tests and Mann–Whitney *U* tests or chi-squared tests and Fisher’s exact tests, independently. Logistic regression was used to determine independent risk factors related to PDX generation. The kappa coefficient value was calculated to measure the agreement of chemotherapy response between PDXs and PAs, while the long-rank test and Kaplan–Meier curves were used to analyze the PFS. All statistical analyses were performed by SPSS Statistics 23.0 (IBM Corporation, Armonk, NY, USA). A *p*-value less than 0.05 on the bilateral test was statistically significant.

## Results

### Generation of the EOC-PDX Models

This consecutive cohort included 130 cases of high-grade serous ovarian cancer (HGSOC), 12 cases of endometrioid ovarian cancer (EC), 11 cases of clear cell ovarian cancer (CC), 3 cases of mucinous ovarian cancer, and two cases of low-grade serous ovarian cancer (LGSOC), 92 (58.23%) of which were successfully developed into PDXs. The characteristics of the patients and PDXs are detailed in **Supplementary Table S1**. The engraftment rates were similar among different pathologic types (*p* = 0.906), and primary and recurrent status did not affect engraftment rates (56.20% *vs.* 71.43%, *p* = 0.188).

**Figure 1:**
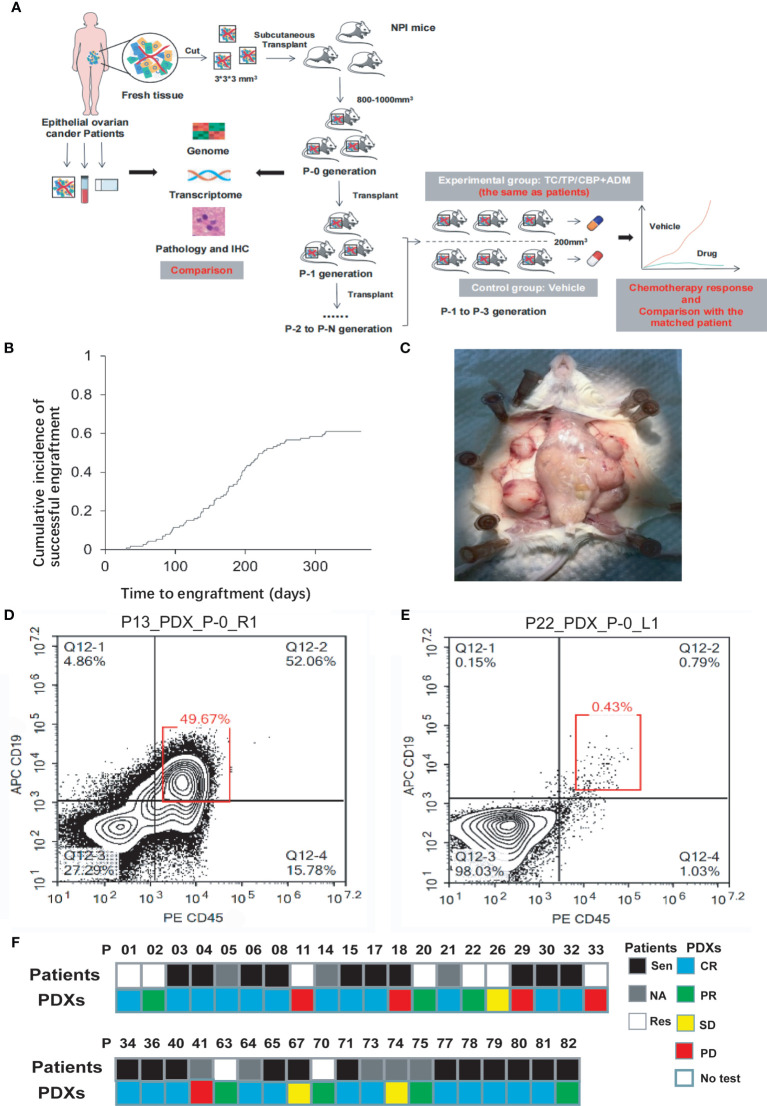
**(A)** The protocol of whole experiment. **(B)** The successful engraftment rate increased over time and was 61.1% 1 year after subcutaneous implantation into mice. **(C)** This panel shows a representative image of the gross appearance of EOC-PDX tumors. Four sites of engrafted tumors (white triangle) have grown to be harvest. **(D, E)** Whether human lymphoma existed in PDX models was quickly checked according to the proportion of CD19+CD45+ cells (red box) via flow cytometry. A proportion of <1% suggested a “clean” PDX model without the development of lymphoma. **(F)** Comparison of chemotherapy efficacy between patients and paired PDX models in 39 patients. The chemotherapy regimens were identical. NPI, NOD-Prkdcem1Idmo-Il2rgem2Idmo; TC, paclitaxel/carboplatin; TP, paclitaxel/cisplatin; CBP, carboplatin; ADM, doxorubicin; Sen, Sensitivity; Res, resistance; CR, complete remission; PR, partial remission; SD, stable disease; PD, progressive disease.

**Figure 1A** depicts the experimental protocol. **Figure 1B** shows the cumulative incidence of the engraftment rate over time. For the P-0 models, the median establishment period was 159 ± 69 days (most were established within 3–6 months), but the P-1 and P-2 models took 93 and 70 days, respectively, which were significantly shorter than the time needed for the P-0 model (*p* < 0.0001). The site and size of the tumor in an established model are shown in **Figure 1C**. Several PDXs from P22, P66, P67, and P74 developed human lymphoma and were excluded from further experiments (examples are shown in **Figure 1D, E**). Chemotherapy sensitivity tests were performed on some PDXs(**Figure 1F**), which will be detailed later.

### Histopathological Similarity Between Patients and PDXs

**Figure 2:**
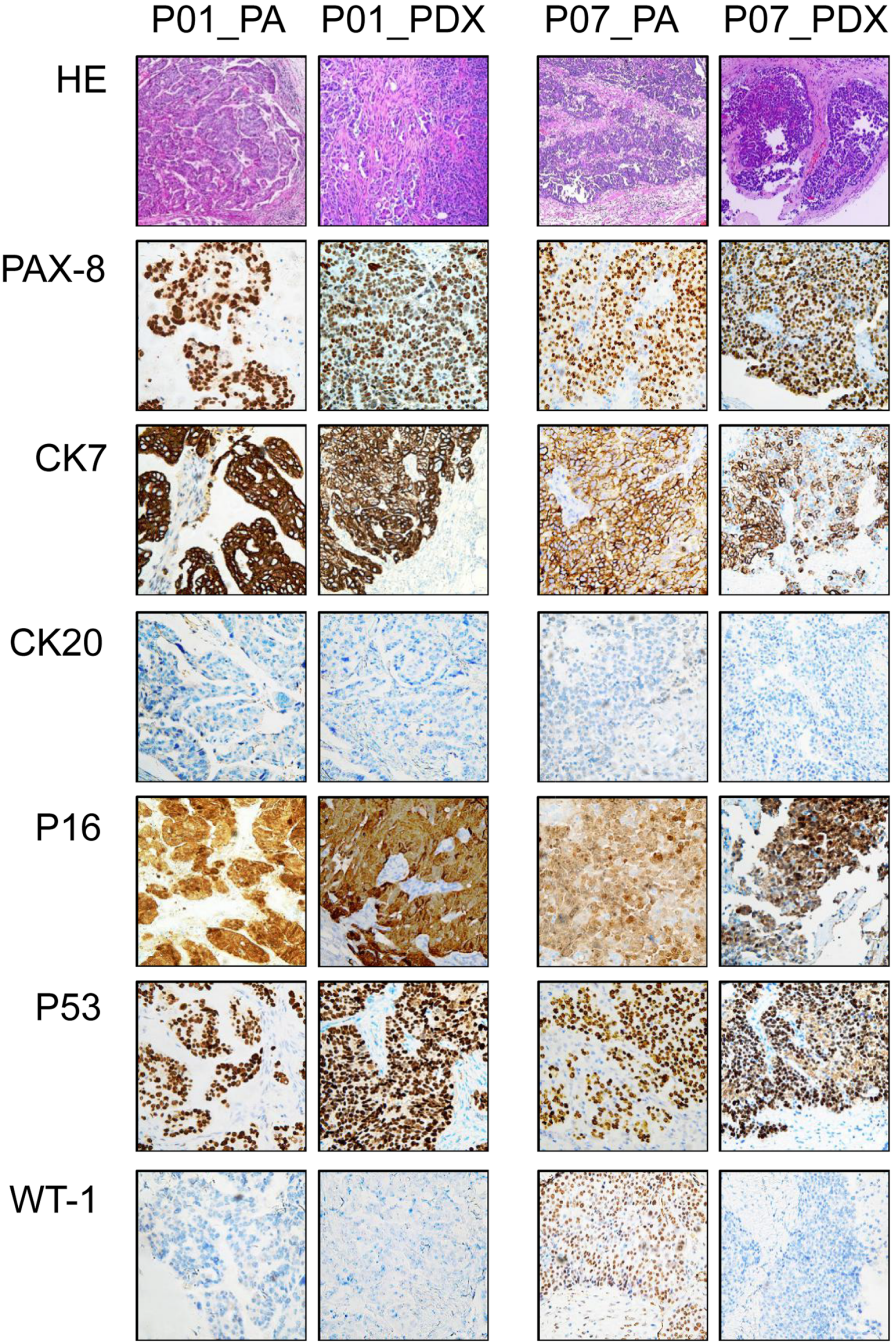
Representative immunohistochemical appearances of PA and PDX tumors. This figure shows immunohistochemical results of paired samples from PA of P01 and P07 patients (both high-grade serous ovarian cancer) and the corresponding PDX models. Primary antibodies used in immunohistochemistry are labeled on the left. PA, patient tumor; PDX, patient-derived xenograft.

HE staining showed similar morphology between the PDXs and original PA tumors, as shown in **Figure 2** (two representative cases, P01 and P07): complex patterns of cystic, papillary, and solid growth were maintained in the PDXs, although they tended to have a higher degree of cytologic atypia than PA in a few cases. IHC was further performed in 10 HGSOC cases to determine whether the PDX tumors had similar protein expression patterns as PA (**Figure 2**, **Supplementary Table S2**). The expression of PAX-8, CK7, WT-1, CK20, P16, P53, ER, and PR was highly consistent in the matched PDX and PA tissues, despite a slightly different WT-1 status (P03, P05–P08).

### Genome Similarity Between Patients and PDXs

**Figure 3:**
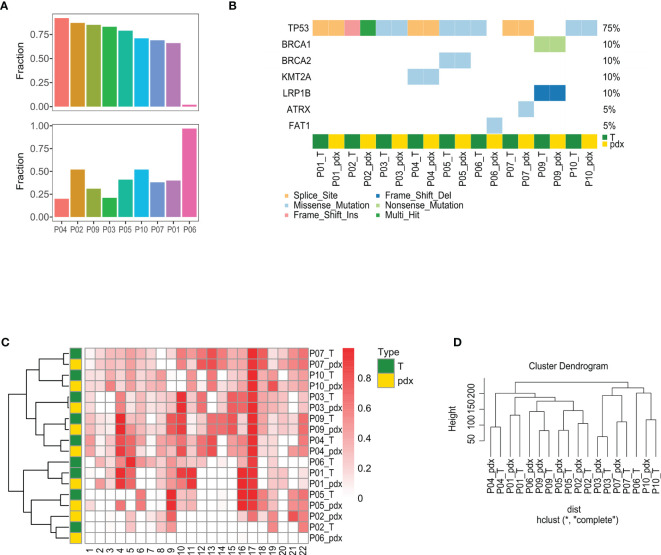
Comparison of mutational features between PA and PDX tumors. **(A)** This figure summarizes the mutation calling results of PA and PDX samples, highlighting the variant classification distributions within a sample type, SNV class, variants per sample, and the most mutated genes. **(B)** Within SMG identified in a pair of PA and PDX tumors, approximately half are shared by both samples, except the paired samples of patient #59. **(C)** Top SMGs (red square) are demonstrated in 10 pairs of PA and PDX tumors. **(D)** This heat map shows the LOH proportion on each chromosome (numbered at the bottom) in each sample. Darker red square represents a higher frequency of LOH. PA and the corresponding PDX tumors have a similar LOH pattern. PA, patient tumor; PDX, patient-derived xenograft; SNV, single-nucleotide variant; SMG, significantly mutated gene; LOH, loss of heterozygosity.

WES was performed for 10 paired P-0 PDXs and PAs for genome comparisons, but P09 was not included due to degradation. The sequencing run generated a 194–389 read depth (**Supplementary Table S3**). Median somatic variants were 81.5 and 79.5 in PA and PDX samples, respectively, and they shared a similar exonic mutation distribution and similar single-nucleotide variant (SNV) types (**Supplementary Figure S1A**). Mutation levels, driving mutation types, LOH status, and gene copy number were then analyzed, and good similarity was observed (**Figure 3**).

Approximately 75% of PA mutations were also detected in the corresponding PDXs, except for P06, indicating the high consistency of mutation levels, but those same mutations accounted for only approximately 35% of PDX mutations (**Figure 3A**). Although the same mutations in P06 were found in only approximately 5%, they accounted for more than 90% of PDX mutations. To clarify whether the mutation types detected in PAs were still maintained in PDXs, the mutation type of disease-driving genes characterized in HGSOC was analyzed. Cluster analysis showed that seven patients had homogeneous mutations, although one patient had mutation loss (P06) and one had a mutation type change (P02) (**Figure 3B**). LOH is a sign of gene scarring, which is permanent in both time and individual characteristics, so we further compared whether the LOH status was consistent between PAs and PDXs. The heat map illustrates the proportion of LOH on each chromosome from the different samples, with chromosome 17 showing a higher LOH ratio, and cluster analysis showed that the LOH status was highly consistent, except for P06 (**Figure 3C**). This concordance was also seen in the gene copy number (**Figure 3D**).

### Altered Transcriptomes Between Patients and PDXs

**Figure 4:**
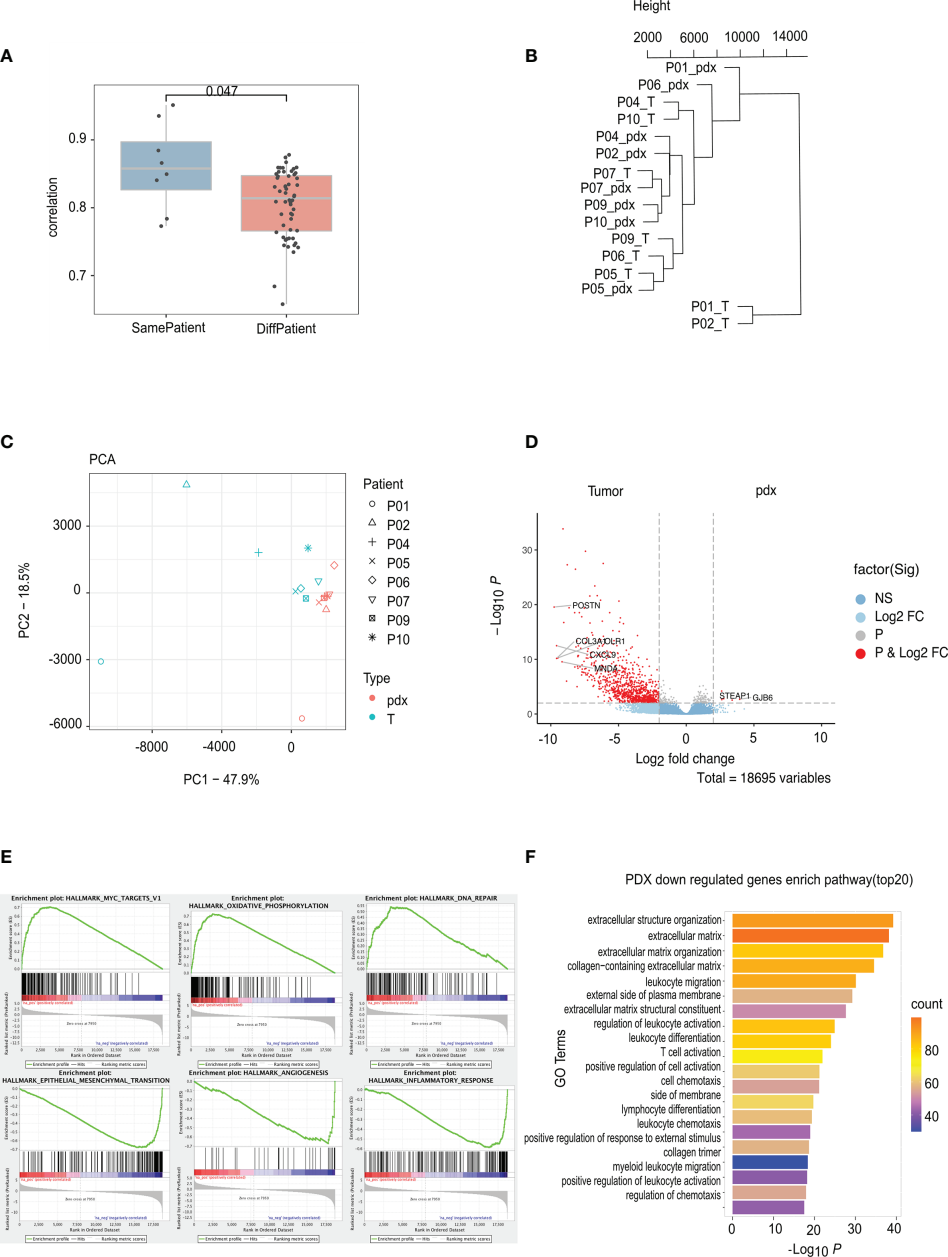
RNA sequencing analyses of PA and PDX tumors. **(A)** The correlation between PA and PDX samples from the same patient is significantly higher than that between samples from different patients. **(B)** This phylogenetic tree depicts relationships of nine PA samples and their corresponding PDX samples at the RNA level. There is no clustering tendency of PDX and its original PA samples. **(C)** The technique of PCA was used to simplify the complexity in high-dimensional data of RNA sequencing and to visualize clustering of samples. PDX and PA samples are roughly separated by the dotted line, indicating an altered mode of transcription due to implantation. **(D)** DEGs volcanic figure. **(E)** PDX overexpressed genes clustered in Myc, oxidative phosphorylation, and DNA damage repair pathways, while low-expressed genes clustered in interstitial transformation, angiogenesis, and inflammatory responses. **(F)** GO analysis of low expression gene of PDX. PA, patient tumor; PDX, patient-derived xenograft; PCA. principal component analysis; DEGs, different expression genes.

Total RNA from nine paired samples was sequenced for transcriptome comparisons. **Figure 4A** displays the transcriptome similarity; the correlation between matched PAs and PDXs was significantly higher than that between unmatched PAs and PDXs (*p* = 0.047), but the average was close, and the scope overlapped to a great extent. As a result, cluster analysis and principal component analysis (PCA) were used to further explore the discrepancy: the PDXs were deficient in terms of the clustering tendency with corresponding PAs; instead, PDXs and PAs were clustered separately (**Figures 4B, C**). DEG analysis and pathway enrichment analysis were then performed to explore these differences: 17,971 DEGs were identified, primarily downregulated in PDXs, and only 6 were upregulated (**Figure 4D**). The former was enriched in the epithelial–mesenchymal transformation, angiogenesis, and inflammatory response pathways, and the latter was enriched in the Myc, oxidative phosphorylation, and DNA damage repair pathways (**Figure 4E**). Similarly, in the Gene Ontology (GO) analysis, the significantly downregulated genes in PDXs were enriched in extracellular mechanisms and inflammatory pathways (**Figure 4F**).

### Chemotherapy Response of Patients and PDXs

Reflecting the treatment efficacy of PAs is the premise of clinical application for PDXs, so the first-line chemotherapy responses of 39 PDXs were compared with matched PAs. All chemotherapy regimens administered to the PDXs were the same as those administered to PAs. Most PAs received standard first-line chemotherapy with paclitaxel and carboplatin (TC) or paclitaxel and cisplatin (TP), and several received carboplatin and doxorubicin (**Supplementary Table S5**). The mean PFS of PAs was 12.77 months. A total of 70.97% (22/31) of patients exhibited platinum sensitivity, except for eight missed patients, and 81.82% (18/22) of PDXs showed mCR, indicating excellent consistency in the drug response between PAs and PDX models (kappa = 0.644, *p <* 0.001) (**Figure 1E**).

**Figure 5:**
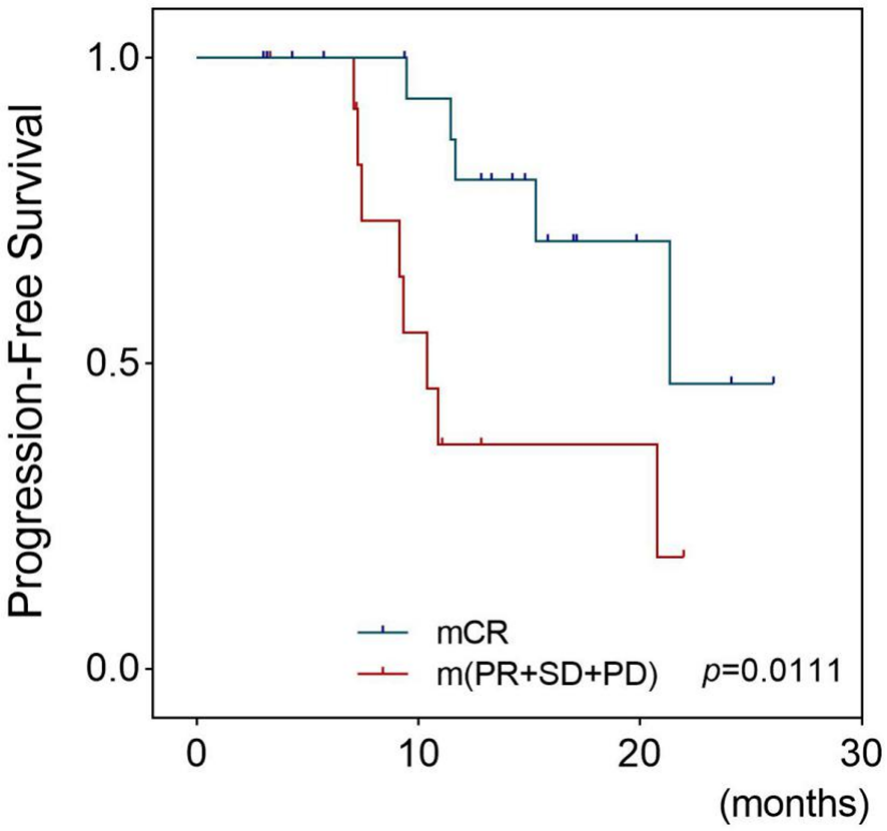
Survival outcomes of patients whose PDX models had different platinum responses. This figure shows the Kaplan–Meier plots for progression-free survival of EOC patients who received chemotherapy based on platinum. Patients were divided into two groups: the mCR group whose corresponding models achieved mCR and the m(PR+SD+PD) group whose models were evaluated as mPR, mSD, or mPD. The Tick marks indicate censored data. *p*-values were estimated with the use of log-rank test based on the univariate analysis. PDX, patient-derived xenograft; EOC, epithelial ovarian cancer; mCR, modified complete response; mPR, modified partial response; mSD, modified stable disease; mPD, modified progressive disease.

The patients were then divided into two groups based on the PDX chemotherapy response: the mCR group (24 cases) and the m(PR+SD+PD) group (15 cases). The estimated mean PFS was 21.40 months and 13.51 months for the mCR group and m(PR+SD+PD) group, respectively, suggesting that the mCR of the PDXs was associated with a longer PFS in clinical patients (*p* = 0.003) (**Figure 5**).

### Factors Associated With Engraftment Rates

Considering that primary and recurrent ovarian cancer may have diverse clinical characteristics, we analyzed factors affecting engraftment rates separately, as shown in **Tables 1**, **2**. A low Ki-67 index and receiving NACT were independent risk factors for the failure of engraftment in primary EOC patients (*p* = 0.013 and 0.045, respectively), while no independent risk factor was found in recurrent patients. A higher Ki-67 index and higher LOH status in NACT tumors than in non-NACT tumors may be associated with this phenomenon (*p* = 0.018 and 0.014, respectively) (**Supplementary Figures B**–**F**).

**Table 1:** Factors affecting engraftment rates in primary EOC-PDX models.

Factors	*N*	Success	Failure	*p-*value	
				Univariate analysis	Multivariate analysis
**Age (years)**	137	56.68 ± 9.26	54.70 ± 10.52	0.245	
**CA125 (U/ml)**	137	400.6	316.75	0.854	
		(121.1, 1,012)	(65.88, 847.90)		
**NACT**				0.018	0.045
Yes	62	28	34		
No	75	49	26		
**Surgical Satisfaction**				0.289	0.283
R0	88	46	42		
R1	32	21	11		
Non-satisfaction	1	1	0		
**Pathological type**				0.259	0.772
Type I EOC	24	11	13		
Type II EOC	113	66	47		
**Cancer grade**				0.179	0.203
Low–intermediate	9	3	6		
High	128	74	54		
**FIGO stage**				0.589	
II	13	6	7		
III	95	56	39		
IV	29	15	14		
**Ki-67(positive%)**	127	60%	40%	<0.001	0.013
		(40%, 80%)	(21.25%, 60%)		
***TP*53**				0.353	
Wild type	31	15	16		
Mutant type	102	59	43		
***BRCA*1/2**				0.445	
Wild type	52	31	21		
Mutant type	22	11	11		
**Response to Platinum**				0.978	
Sensitive	79	45	34		
Negative	28	16	12		

Age is described as mean ± SD, and CA125 and Ki-67 level are described as median (quartiles).

EOC, epithelial ovarian cancer; PDX, patient-derived xenograft; NACT, neoadjuvant chemotherapy; FIGO, International Federation of Gynecology and Obstetrics.

**Table 2:** Factors affecting engraftment rates in recurrent EOC-PDX models.

Factors	*N*	Success	Failure	*p-*value	
				Univariate analysis	Multivariate analysis
**Age (years)**	21	52.87 ± 9.59	59.5 ± 6.55	0.115	0.187
**Types of recurrence**				1.00	
Platinum sensitive	13	9	4		
Platinum resistance	8	6	2		
**CA125 (U/ml)**	21	90.5	115.3	0.448	
		(35.45, 630.8)	(81.23, 221.6)		
**Pathological type**				0.526	
Type I EOC	3	3	0		
Type II EOC	18	12	6		
**Cancer grade**				1.00	
Low–intermediate	1	1	0		
High	20	14	6		
**FIGO stage**				0.327	
II	1	1	0		
III	17	11	6		
IV	3	3	0		
**Ki-67(positive%)**	21	60%	40%	0.445	
		(40%, 75%)	(28.75%, 70%)		
***TP*53**				0.079	0.999
Wild type	2	0	2		
Mutant type	18	14	4		
***BRCA*1/2**				1.00	
Wild type	4	3	1		
Mutant type	4	3	1		

Age is described as mean ± SD, and CA125 and Ki-67 level are described as median (quartiles).

EOC, epithelial ovarian cancer; PDX, patient-derived xenograft; NACT, neoadjuvant chemotherapy; FIGO, International Federation of Gynecology and Obstetrics.

## Discussion

This study successfully constructed large-scale ROC-PDX models and verified the consistency of paired PDXs’and patients’ tumors at the pathology, molecular level, chemotherapy response, and clinical outcome despite the differences in the transcriptome, demonstrating that the PDX model could be a preclinical tool for personalized treatment.

A highlight of this paper is the consecutive cohort and a large number of patients enrolled in modeling, which can mimic the clinical situation of EOC to the greatest extent possible and can guarantee the authenticity and credibility of the research results—the patients’ pathological distribution and the percentage of platinum-sensitive patients resembled the clinic. Diverse protocols such as intraperitoneal or orthotopic transplantation and cell suspension or fine-needle injection have increased the inaccuracy of cancer tissue characteristics and difficulty of treatment assessments (18, 22), so we only used subcutaneous transplantation with 3 × 3 × 3 mm^3^ tissue blocks. Moreover, the engraftment success rate (58.23%) was comparable to other research (18.5%–85.3%) (18, 20–24).

The tumorigenesis period in this study was longer than that in Meng et al. (21–130 days) (25) and Wu et al. (21–51 days) (23). Nevertheless, the former study regarded 66 mm^3^ rather than 800 mm^3^ as the threshold of successful modeling, and it took 200 days for the tumor volume to reach 800 mm^3^, longer than our study. The engraftment rate was low (18.52%) in the latter research, and the passage was not stable (60% of P1 failed to form P2). Other studies required 12 months at most for EOC-PDX model establishment (18, 20, 21). What needs to be emphasized is that the modeling time is still less than the median PFS (12 months) of PAs, implying that there is enough time to find platinum-resistant patients and screen second-line chemotherapy regimens with PDX models.

Notably, an increasing number of studies have begun to challenge the reliability of PDX models (26–28). Ben-David et al. (4) compared the genomes between PA and successive PDX tumors and observed that the CNV pattern of PDXs gradually trended away from the PAs due to environmental selection in mice. Sato et al. (3) found the same phenomenon and noted that even driving mutations could be converted in PDXs. Liu et al. (26) observed different transcriptomes in PDXs and assumed that the replacement of human stroma and inflammatory components by mouse tissue might trigger this variation, but they did not assess whether DEGs would alter treatment effects.

Given this, WES and RNA-seq were performed in this study to compare the genome and transcriptome between PDXs and PAs, and most PDXs were found to recapitulate the genomic characteristics of PAs. However, we still found a tiny discrepancy: new mutations were detected in PDXs, with PA-derived mutations accounting for only approximately 35% of PDX mutations, and the mutant types of a few cancer-driving genes were transformed. The significant genomic differences between the PA and PDX tumors observed in P06 may be due to the imbalanced growth of primary tumor cells or the loss of mutations because more than 90% of mutations derived from PA and TP53 mutations were lost in the PDX model. The transcriptome of PDXs was significantly distinctive from that of PAs in the cluster analysis. Enrichment analysis of DEGs showed that the interstitial and immune-related pathways were inhibited, and the pathways related to proliferation and survival were activated, implying that changes in RNA levels may be due to heterogeneity within the tumor and the change in mice, and can benefit tumor cell survival.

Furthermore, chemosensitivity tests were performed to determine whether this differential expression would affect drug efficiency. The chemotherapy regimens used in PDXs were the same as those used in the paired PAs, and the response was compared in a one-to-one manner, which is an improvement over other studies (18–20, 24). The PDXs showed a similar platinum response to the paired PAs, indicating that nondriving DEGs would not affect drug sensitivity, strongly supporting the application of PDXs to validate drug sensitivity *in vivo*. Furthermore, patients whose PDXs showed a better chemotherapeutic response had a better prognosis, such as in Topp et al. (24), implying that patients with platinum resistance in the PDXs need to begin second-line chemotherapy tests as soon as possible.

The factors affecting the engraftment rate remain unclear in EOC, but some researchers have suggested that it is not associated with the FIGO stage or WHO cancer grade (18, 23). Our results are consistent with these findings; however, the role of pathology remains controversial. Wu et al. (23) found that ovarian germ cell tumors had the highest engraftment rates (100%), but limited cases may undermine the reliability of this result since there was no difference observed between borderline tumors and malignancy in PDX construction. Ricci et al. (18) failed to find any influence of pathological subtypes on modeling, as suggested in this paper. In addition, Ki-67, a cell proliferation index, and NACT were found to influence the engraftment rate of primary patients. The reduced Ki-67 index and LOH frequency in NACT patients confirmed that further.

One limitation of this study is that only a few type I EOCs (28 cases) were included. Type I and type II EOCs have different biological and molecular characteristics (31), which might affect tumorigenicity in PDXs, but we failed to find it. However, this could be related to its low incidence and may be further explored by expanding the sample. In addition, economic costs and the time needed to generate PDX models (3–6 months) may limit their utilization. Nevertheless, the time needed for model establishment allows us to identify platinum-resistant patients and conduct second-line chemotherapeutic screening, as the mean PFS for PAs was approximately 12 months.

In conclusion, we established robust EOC-PDX models, recapitulating patients’ pathology, genome, and protein expression and predicting patients’ treatment response and prognosis, so PDXs are a promising and reliable preclinical tool for personalized and precise treatment.

## Data Availability Statement

The raw data supporting the conclusions of this article will be made available by the authors, without undue reservation.

## Ethics Statement

The patients/participants provided their written informed consent to participate in this study. The animal study was reviewed and approved by Institutional Review Board of Peking Union Medical College Hospital (PUMCH).

## Author Contributions

Data curation: JC, SL, and YY. Formal analysis: JC, SL, YD, and SP. Funding acquisition: LP. Investigation: JC, XP, and YD. Methodology: YJ, CQ, YD, and SP. Resources: YL, YG, WW, JY, YS, Y-XW, MQ, and YC. Software: HL. Supervision: YJ and LP. Visualization: YY. Writing—original draft: JC and SL. Writing—review and editing: YJ, YD, SP, and LP. All authors contributed to the article and approved the submitted version.

## Funding

This project was supported by the CAMS Innovation Fund for Medical Sciences (CIFMS-2017-I2M-1-002), the fund of the National Key R&D Program of China 2016YFC1303700 (Affiliated project 2016YFC1303701), and the Fund of National Natural Science Foundation of China (30772315, 81572564, 31171416 and 31471383). This project was also supported by the fund of the Beijing Municipal Science & Technology Commission (Z181100001918043) from Beijing IDMO Co., Ltd. This study received funding from Beijing IDMO Co., Ltd.(the Beijing Municipal Science & Technology Commission (Z181100001918043)), which was not involved in the study design, collection, analysis, interpretation of data, the writing of this article, or the decision to submit it for publication.

## Conflict of Interest

Authors CQ and SP were employed by the company Beijing IDMO Co., Ltd. Authors XP, HL, and YD were employed by the company Precision Scientific (Beijing) Co., Ltd.

The remaining authors declare that the research was conducted in the absence of any commercial or financial relationships that could be construed as a potential conflict of interest.

## Publisher’s Note

All claims expressed in this article are solely those of the authors and do not necessarily represent those of their affiliated organizations, or those of the publisher, the editors and the reviewers. Any product that may be evaluated in this article, or claim that may be made by its manufacturer, is not guaranteed or endorsed by the publisher.
