# Supplementary material for: Patient-Derived Xenografts Are a Reliable Preclinical Model for the Personalized Treatment of Epithelial Ovarian Cancer
Source: Front Oncol. 2021 Oct 4;11:744256. doi: 10.3389/fonc.2021.744256 (PMC8522495; doi:10.3389/fonc.2021.744256)
Supplement: Supplementary file 1 [file DataSheet_1.docx]

| **A**  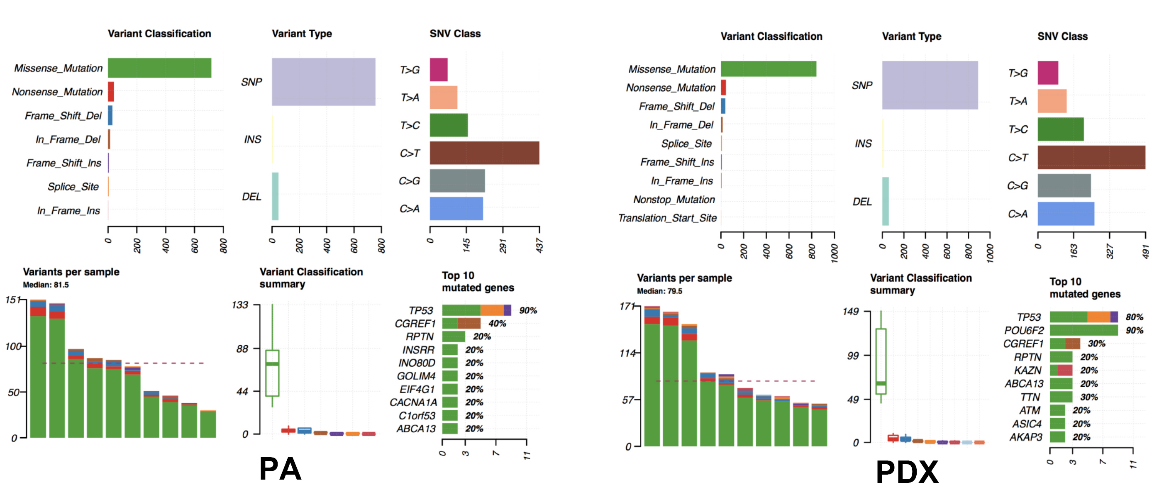  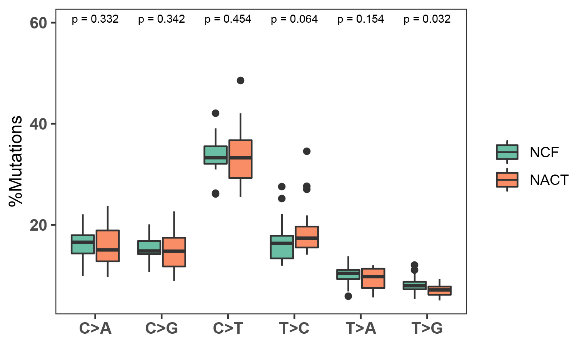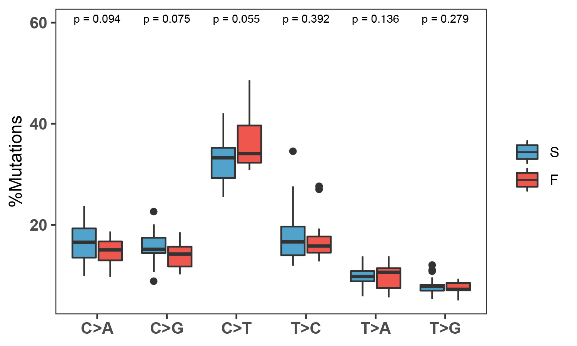**B C**      **D E F**    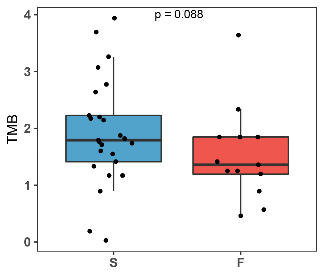 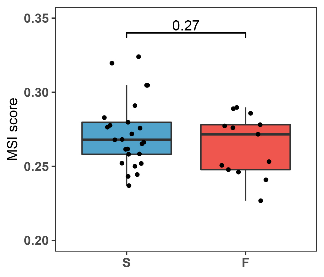 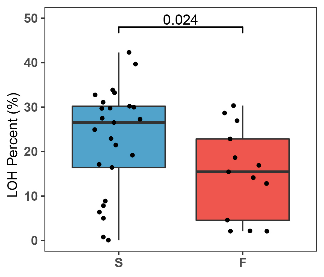  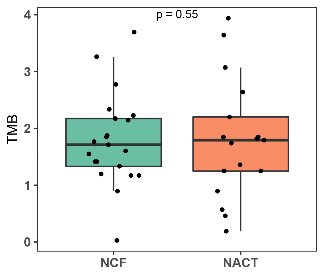 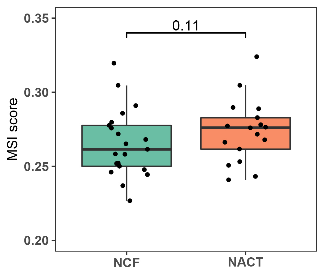 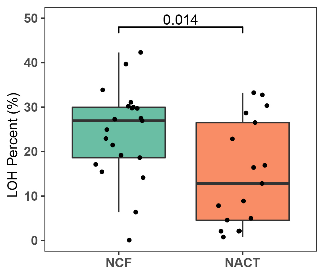 |
| --- |
| **Supplementary Figure 1** Mutational features associated to the engraftment rate  Panel A summarizes the mutation calling results of PA and PDX samples, highlighting the variant classification distributions within a sample type, SNV class, variants per sample and the most mutated genes. In panel B to panel F, the proportion of base transitions and transversions (TiTv), tumor mutation burden (TMB), microsatellite instability (MSI) score and the proportion of loss of heterozygosity (LOH) are respectively compared between groups. The blue bar represents tumors that failed in generation of PDXs. The rea bar represents tumor that successfully engrafted. The green bar represents neoadjuvant chemotherapy-free (NCF) tumors. The orange bar represents tumors treated with neoadjuvant chemotherapy (NACT). |
